# Polyphyllin VII Induces an Autophagic Cell Death by Activation of the JNK Pathway and Inhibition of PI3K/AKT/mTOR Pathway in HepG2 Cells

**DOI:** 10.1371/journal.pone.0147405

**Published:** 2016-01-25

**Authors:** Chao Zhang, Xuejing Jia, Kai Wang, Jiaolin Bao, Peng Li, Meiwan Chen, Jian-Bo Wan, Huanxing Su, Zhinan Mei, Chengwei He

**Affiliations:** 1 State Key Laboratory of Quality Research in Chinese Medicine, Institute of Chinese Medical Sciences, University of Macau, Macao 999078, China; 2 College of Pharmacy, South Central University for Nationalities, Wuhan 430074, China; National Institute of technology Rourkela, INDIA

## Abstract

Polyphyllin VII (PP7), a pennogenyl saponin isolated from *Rhizoma Paridis*, exhibited strong anticancer activities in various cancer types. Previous studies found that PP7 induced apoptotic cell death in human hepatoblastoma cancer (HepG2) cells. In the present study, we investigated whether PP7 could induce autophagy and its role in PP7-induced cell death, and elucidated its mechanisms. PP7 induced a robust autophagy in HepG2 cells as demonstrated by the conversion of LC3B-I to LC3B-II, degradation of P62, formation of punctate LC3-positive structures, and autophagic vacuoles tested by western blot analysis or InCell 2000 confocal microscope. Inhibition of autophagy by treating cells with autophagy inhibitor (chloroquine) abolished the cell death caused by PP7, indicating that PP7 induced an autophagic cell death in HepG2 cells. C-Jun N-terminal kinase (JNK) was activated after treatment with PP7 and pretreatment with SP600125, a JNK inhibitor, reversed PP7-induced autophagy and cell death, suggesting that JNK plays a critical role in autophagy caused by PP7. Furthermore, our study demonstrated that PP7 increased the phosphorylation of AMPK and Bcl-2, and inhibited the phosphorylation of PI3K, AKT and mTOR, suggesting their roles in the PP7-induced autophagy. This is the first report that PP7 induces an autophagic cell death in HepG2 cells via inhibition of PI3K/AKT/mTOR, and activation of JNK pathway, which induces phosphorylation of Bcl-2 and dissociation of Beclin-1 from Beclin-1/Bcl-2 complex, leading to induction of autophagy.

## Introduction

Hepatocellular carcinoma (HCC) is the third leading cause of cancer-related death worldwide. Surgical resection, the major therapeutic strategy for HCC, is only effective for 15% to 25% early stage of HCC patients. Most HCC patients of late-stage die within a few months after diagnosis due to poor response to chemotherapy and radiotherapy [1]. There is still no effective therapeutic and preventive agents for HCC patients so far [2]. Therefore, it is imperative to research and develop new agents, such as phytochemicals from plants, for the prevention and treatment of HCC.

The development of HCC is believed to correlate with dysregulation of programmed cell death, which is a basic biological phenomenon playing an important role in cancer treatment [3,4]. Autophagy and apoptosis coordinately regulate cell survival and cell death, and occur simultaneously in cancers [5,6]. Autophagy is a cellular self-digestion process involves degradation of unnecessary or dysfunctional cytoplasmic components through the actions of endogenous lysosomes in response to adverse conditions to maintain the cellular energy supply and homeostasis [7,8]. Numerous studies have demonstrated that autophagy can be stimulated by a variety of stressors, such as oxidative stress, mitochondrial damage, nutrient deprivation and exogenous chemicals [9]. Chemotherapeutic agents often activate autophagy in cancer cells. However, many autophagy-modulating agents also have been identified as potential cancer therapeutic agents [10], such as rapamycin and chloroquine [11]. Therefore, autophagy is emerging as an important target in intervention and treatment of cancers [12], and targeting autophagic signaling pathways provides a novel strategy for drug discovery and new targets for drug development in cancer treatment [11]. Autophagy can act as a protective mechanism to promote cellular survival by degrading damaged proteins and organelles [13]. Autophagy can also trigger autophagic death pathway to promote cell death under certain stress conditions [9,14]. Many anticancer agents, such as rapamycin, tamoxifen and imatinib, induce excessive and sustained autophagy in a number of cancer cells, which eventually trigger autophagic cell death [10,15,16]. Additionally, many agents, such as arsenic trioxide [17] and *Polygonatum cyrtonema* lectin [18], from traditional Chinese medicine (TCM) have also been reported to be able to induce autophagic cell death in cancer therapy. Accumulated evidence suggests that autophagy is implicated in common hepatic diseases including ischemia/reperfusion-induced liver injury, viral hepatitis, and HCC [19].

Previous studies have demonstrated that various cellular signaling pathways including adenosine monophosphate-activated protein kinase (AMPK) and phosphatidylinositol 3-kinase/protein kinase B/mammalian target of rapamycin (PI3K/AKT/mTOR) pathways play a vital role in the process of autophagy [20,21]. PI3K/AKT pathway acts as a positive regulator of the mTOR pathway, which serves as a negative regulator of autophagy in cancer cells [11], and disruption of the PI3K/AKT/mTOR pathway by anticancer agents induces autophagy. AMPK is a key regulator of energy to maintain energy homeostasis and activates autophagy by inhibiting mTOR complex 1 (mTORC1) [21,22]. Additionally, c-Jun N-terminal kinase (JNK) pathway is also involved in the regulation of autophagy of cancer cells in response to pharmacological stress [5,23]. Several studies demonstrated that autophagy was often triggered by inhibiting PI3K/AKT/mTOR pathway concomitant with activating the JNK pathway [6,24]. The autophagy protein Beclin-1, a key component of the autophagosome nucleation complex, can interact with Bcl-2 to form Beclin-1/Bcl-2 complex, which functions as an inhibitor of autophagy [25]. JNK activation can phosphorylate Bcl-2, and then degrade Bcl-2 and dissociate Beclin-1 from Beclin-1/Bcl-2 complex, leading to induction of autophagy [26,27,28,29]. Moreover, JNK activation has been essential for autophagic cell death induced by anticancer agents [27,30].

The rhizome of *Paris polyphylla* var. *yunnanensis*, or called *Rhizoma Paridis*, has long been used in TCM, including cancer therapy [31]. *Rhizoma Paridis* mainly contains steroidal saponins, especially diosgenyl saponins and pennogenyl saponins [32]. Several steroidal saponins possessing anticancer properties against a variety of cancer cells have been isolated and identified from *Rhizoma Paridis* [33]. Polyphyllin VII (PP7), an active pennogenyl saponin derived from *Rhizoma Paridis*, has been demonstrated to have strong anticancer activities via suppression of proliferation, cell cycle arrest, and modulation of drug resistance in a wide variety of human cancer cell lines, including liver cancer HepG2 cells [34], cervical cancer Hela cells [35], adriamycin-resistant breast cancer MCF-7 cells [36], and colorectal cancer HT-29 cells [37]. Previous studies found that PP7 induces apoptosis in human liver cancer HepG2 cells [34]. In this study, we aimed to investigate whether PP7 could induce autophagy and its role in PP7-induced cell death and the underlying molecular mechanisms. We found, for the first time, that PP7 induced autophagic cell death in HepG2 cells by activating JNK and AMPK, and inhibiting PI3K/AKT/mTOR pathway.

## Materials and Methods

### Chemicals and Reagents

Polyphyllin VII (PP7) was kindly provided by Prof. Zhinan Mei (South-Central University for Nationalities). The chemical structure of PP7 was determined by NMR and ESIMS on a BRUKER DRX-500 spectrometer and an Agilent G6230 TOF mass spectrometer, respectively. The results of NMR (S1 Table, S1 and S2 Figs) and ESIMS (S3 Fig) for PP7 are identical to that published in previous reports [38,39]. Dulbecco’s modified Eagle’s medium (DMEM) was obtained from Gibco (Carlsbad, CA, USA). Fetal calf serum (FCS) was purchased from Tianhang Biotech (Hangzhou, Zhejiang, China). SP600125 was obtained from Beyotime (Nanjing, Jiangsu, China). Chloroquine (CQ), monodansylcadaverine (MDC) and acridine orange (AO) were purchased from Sigma-Aldrich Co. LLC (Shanghai, China). Primary antibodies against light chain 3 (LC3)-I, LC3-II, Beclin-1, sequestosome-1 (SQSTM1, P62), p53, caspase-3, PI3K, phosphorylated PI3K (P-PI3K/p85 at Tyr458), AKT, P-AKT at Ser473, JNK, P-JNK at Thr183/Tyr185, mTOR, P-mTOR at Ser2448, AMPK, P-AMPK at Thr172, Bcl-2, P-Bcl-2 at Ser87, and GAPDH, and secondly antibodies were purchased from Cell Signaling Technology (Danvers, MA, USA) or Santa Cruz Biotechnology Inc. (Santa Cruz, CA, USA). Lipofectamine^TM^ 2000 was obtained from Invitrogen (Carlsbad, CA, USA). Enhanced green fluorescence protein (EGFP)-LC3 expressing plasmid, pEGFP-LC3, was purchased from Addgene (Cambride, MA, USA). The enhanced chemiluminescence (ECL) detection kit was obtained from BD Biosciences (Bedford, MA, USA). All other chemicals of analytical grade were purchased from local sources.

### Cell culture and treatments

Human hepatocellular carcinoma HepG2 cells were obtained from American Type Culture Collection (Manassas, VA, USA). Cells were cultured in DMEM supplemented with 10% heat-inactivated FCS and 1% antibiotics (100 units/mL penicillin-streptomycin), in a humidified atmosphere of 5% CO_2_ at 37°C. Exponentially growing cells were used in the experiments. For all in vitro assays, PP7 was dissolved in dimethyl sulfoxide (DMSO) to make a stock solution. The working solutions were freshly diluted in the basal medium, at a final DMSO concentration of less than 0.1%.

### Cell viability assay

The cytotoxicity of PP7 was measured using 3-(4,5-dimethylthiazol-2-yl)-2,5- diphenyltetrazolium bromide (MTT) colorimetric assay [40]. Briefly, HepG2 cells (1 × 10^4^ cells/well) were seeded in 96-well plates. After complete adhesion, the culture medium was removed and then different dilutions of PP7 or DMSO were added and incubated further for 24, 48, or 72 h. Subsequently, the treated cells were then incubated in freshly DMEM medium containing 1 mg/mL MTT at 37°C. After additional 4 h, the supernatants were replaced with DMSO to solubilize the formazan precipitates. The absorbance at 570 nm was measured with a microplate reader (BioTek, Winooski, VT, USA). The relative viability of treated cells was expressed as percentage of control untreated cells. The morphology of PP7-treated cells was observed and photographed using an AxioCam HRC CCD camera (Carl Zeiss, Oberkochen, Germany) under light microscope.

### MDC and AO staining

Both MDC and AO have been proposed as a tracer for autophagic vacuoles [41]. HepG2 cells cultured in 96-well plates were either treated with indicated concentrations of PP7 or left untreated as control for 24 h at 37°C. Cells were then stained with 50 μM MDC or 1 μg/ml AO in fresh DMEM and incubated for 30 min at 37°C in the dark. After three times washing with phosphate buffered saline, the cells were immediately visualized using the InCell 2000 confocal microscope (GE Biosciences, Piscataway, NJ, USA). Images were captured by visualizing green fluorescence (MDC) and red fluorescence (AO). The mean fluorescence intensities of the cells were measured by the software modules supplied with the InCell 2000.

### GFP-LC3 transient transfection and detection of punctate LC3-positive structures

We detected autophagosome formation by green fluorescence protein (GFP)-LC3 puncta incorporating into autophagic vacuoles [42]. For transient transfection, HepG2 cells were seeded in 96-well plates, and the expression vector for dominant positive LC3 (EGFP-LC3) was introduced into HepG2 cells using Lipofectamine^TM^ 2000 according to the manufacturer’s instructions. The expression vector was transfected 24 h before treatment with 1.32 μM PP7 or left untreated as control for another 24 h at 37°C, and then visualized GFP-LC3 puncta using the InCell 2000 confocal microscope.

### Western blot analysis

For Western blot analysis, HepG2 cells of different treatment groups were collected. Cell extracts were lysed in radio immunoprecipitation assay lysis buffer. Equal amounts of proteins from each group were separated using 5–12% sodium dodecylsulfate polyacrylamide gel electrophoresis, and electrotransferred to polyvinylidene fluoride membranes. The membranes were blocked with 5% nonfat milk in Tris-buffered saline buffer and incubated overnight at 4°C with primary antibodies, followed by incubation with the corresponding secondary antibodies for 1 h at room temperature. Signals were developed using an ECL detection kit. Densitometric measurement of band intensity was performed with the Image Lab Software (Bio-Rad, Hercules, CA, USA).

### Inhibitor Treatment

To clarify the roles of signaling pathways in PP7-induced autophagy in HepG2 cells, the cells were pretreated with the following inhibitors individually before the treatment of PP7 (1.32 μM): 20 μM SP600125 (JNK specific inhibitor), or 10 μM CQ (autophagy inhibitor). The cells were then subjected to the measurement of autophagy-related protein levels by Western blotting as described above.

### Statistical analysis

Data were given as the means ± standard deviation for each group, and analyzed for statistical significance using one-way analysis of variance with Tukey post hoc analysis using GraphPad Prism 5.0 software package (GraphPad Software, San Diego, CA, USA). Differences were considered statistically significant for (*) *P* < 0.05 and (**) *P* < 0.01.

## Results

### PP7 inhibited the proliferation of HepG2 cells

**Fig 1:**
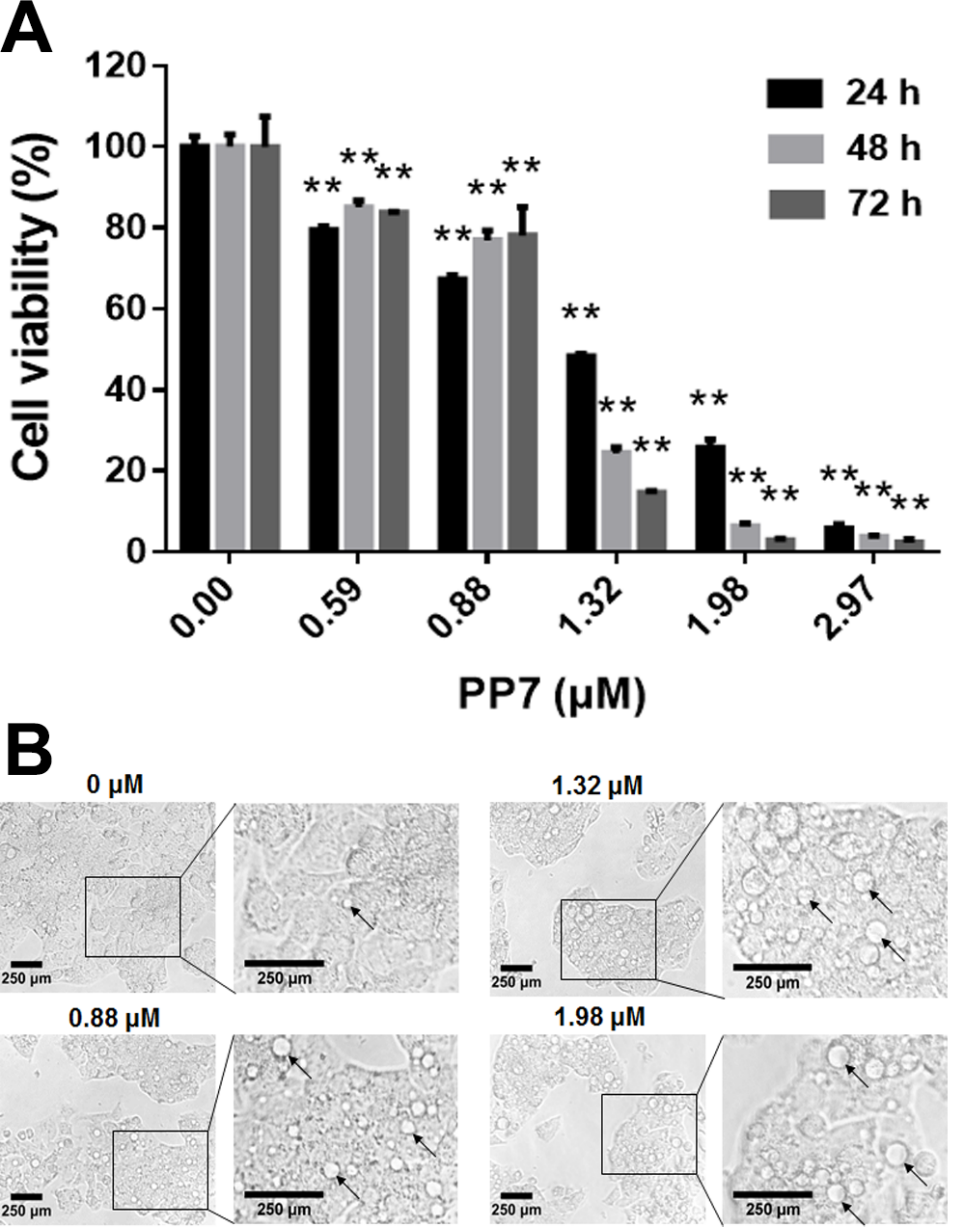
PP7 inhibits the proliferation of HepG2 cells. (A) Cells were treated with indicated concentrations of PP7 for 24, 48 or 72 h. The cell viability was analyzed by MTT assay. Values represent the mean ± SD of at least three independent experiments. ** *P* < 0.01, versus control groups. (B) Morphology of HepG2 cells treatment with PP7 or vehicle control for 24 h was observed under light microscopy (10X objective). Scale bars represent 250 μm.

To examine the anti-proliferation of PP7, HepG2 cells were treated with PP7 at concentrations from 0.59 μM to 2.97 μM for 24, 48, and 72 h, and MTT assay was applied to test the cell viability. The results showed that PP7 significantly inhibited the growth of HepG2 cells in a dose-dependent manner, with a 50% inhibitory concentration value of 1.32 ± 0.04 μM after PP7 treatment for 24 h. In addition, prolonged exposure of HepG2 cells to PP7 resulted in an increased growth inhibitory effect (Fig 1A), indicating that PP7 exhibited strong anticancer effect on HepG2 cells in vitro.

### PP7 induced autophagy in HepG2 cells

**Fig 2:**
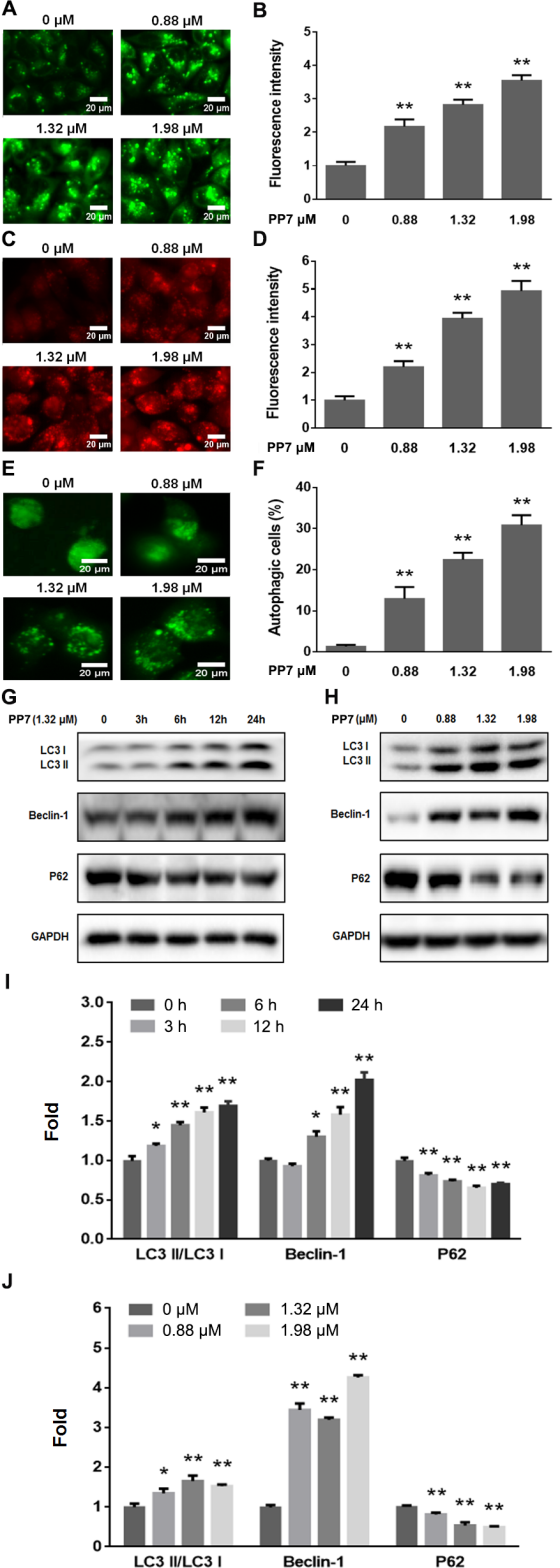
Induction of autophagy by PP7 in HepG2 cells. (A) Monodansylcadaverine (MDC) and (C) Acridine orange (AO) staining. HepG2 cells were treated with vehicle or PP7 for 24 h, stained with MDC and AO, and observed under InCell 2000 confocal microscope (20X objective). The mean fluorescence intensities of MDC (B) and AO (D) in the cells were calculated. (E) GFP-LC3-transfected HepG2 cells were incubated with or without PP7 for 24 h. The GFP-LC3 puncta was observed by InCell 2000 confocal microscope (60X objective). (F) Quantification of autophagic cells (E). Scale bars represent 20 μm. Western blot analysis of the LC3II, Beclin-1 and P62 in HepG2 cells treated with 1.32 μM PP7 for different times (G) or treated with varying concentrations of PP7 for 24 h (H). (I) and (J) were densitometic analysis of (G) and (H), respectively. Data are represented as means ± SD from 3 independent experiments. * *P* < 0.05, ** *P* < 0.01, versus control groups.

To investigate whether PP7 induces autophagy in HepG2 cells, we examined the formation of autophagic vacuoles using the specific fluorescent dyes AO and MDC [41]. Characteristic examples of our observations and quantitative image analysis were shown in Fig 2. The green fluorescence intensities of MDC staining were increased by 117.3, 182.4 and 254.8% when HepG2 cells were treated with 0.88, 1.32 and 1.98 μM of PP7 for 24 h (Fig 2A and 2B). PP7 treatment also resulted in an increased formation of the AO-labeled vacuoles compared to the untreated cells (Fig 2C). The red fluorescence intensity of AO was significantly (*p* < 0.01) increased by PP7 in a dose-dependent manner and reached its maximum intensity when treated the cells with 1.98 μM of PP7 for 24 h (Fig 2D). In addition, we monitored the levels of LC3II conversion (a marker of autophagosomes) and P62 (an indicator of autophagic flux) [42,43]. Western blotting results showed that PP7 increased the protein levels of Beclin-1 and the conversion of LC3I to LC3II, while P62 was decreased after PP7 treatment in an obvious time- and dose-dependent manner (Fig 2G and 2H). Their maximum protein levels were induced by 1.98 μM PP7 for 24 h. Under light microscope, a typical morphological feature of cytoplasmic vacuole accumulation was observed in HepG2 cells after treatment with various concentrations of PP7 for 24 h (Fig 1B). To further confirm PP7-induced autophagy, we transfected HepG2 cells with GFP-LC3 expressing vector and examined whether PP7 could induce GFP-LC3 puncta formation, which is a marker of autophagosomes formation [42]. As shown in Fig 2E, punctate fluorescence (GFP-LC3 dots) was observed in PP7-treated GFP-LC3 expressing HepG2 cells, and PP7 strongly induced autophagic cells in a dose-dependent manner (Fig 2F). Taken together, these data indicated that PP7 induced a robust autophagy in HepG2 cells.

### PP7 induced autophagic cell death in HepG2 Cells

**Fig 3:**
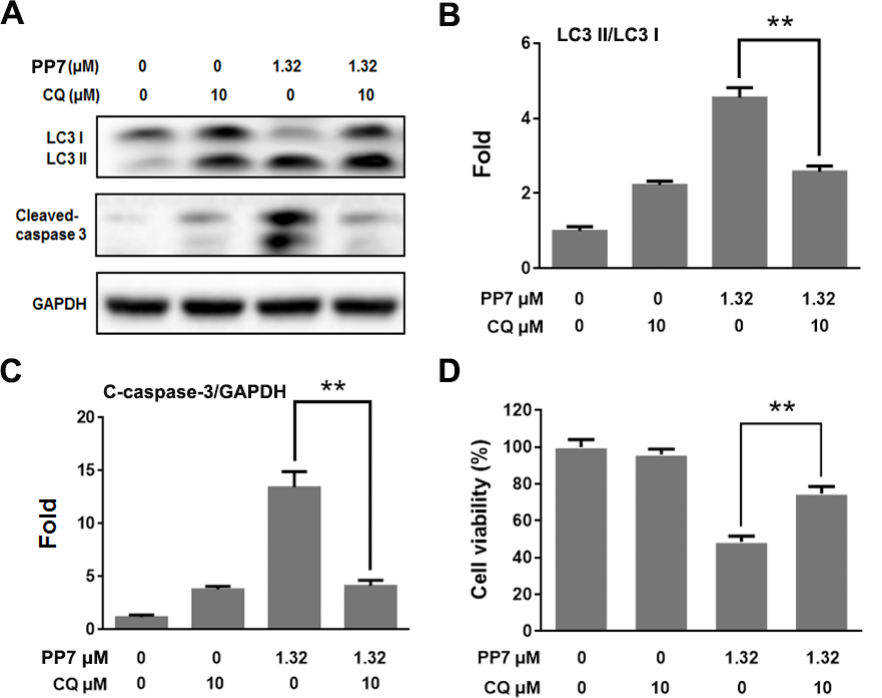
Inhibition of autophagy decreased PP7-induced cell death and apoptosis in HepG2 cells. (A) HepG2 cells were pretreated with 10 μM chloroquine (CQ) for 1 h prior to treatment with 1.32 μM PP7 24 h. The inhibitory effects of CQ on protein levels of LC3II and cleaved caspase-3 were determined by Western blotting. (B) and (C) Densitometic analysis of (A) from three experiments. (D) Pretreatment with 10 μM CQ for 1 h prior to treatment with 1.32 μM PP7 for 24 h. Cell viability was evaluated by MTT assay. Values represent the mean ± SD of at least three independent experiments. ** *P* < 0.01, compared to PP7-treated alone groups.

Natural compound-induced autophagy may be either pro-survival or pro-death in cancer therapy [44]. Therefore, we evaluated the role of autophagy in apoptosis in HepG2 cells induced by PP7. Previous studies have shown that PP7 could increase the level of cleaved caspases-3 in a dose-dependent manner in HepG2 cells [34]. Western blot analysis indicated that PP7 (1.32 μM) could significantly increase the level of cleaved caspase-3, and the protein level of cleaved caspase-3 was obviously decreased via pretreatment with CQ in PP7-treated HepG2 cells (Fig 3A and 3C). MTT results showed that the cell viability was increased from 48.6% (1.32 μM PP7 only) to 74.8% (1.32 μM PP7 + CQ) (Fig 3D). These results revealed that PP7 could induce autophagic cell death in HepG2 cells.

### PP7 induced autophagy via PI3K/AKT/mTOR pathway

**Fig 4:**
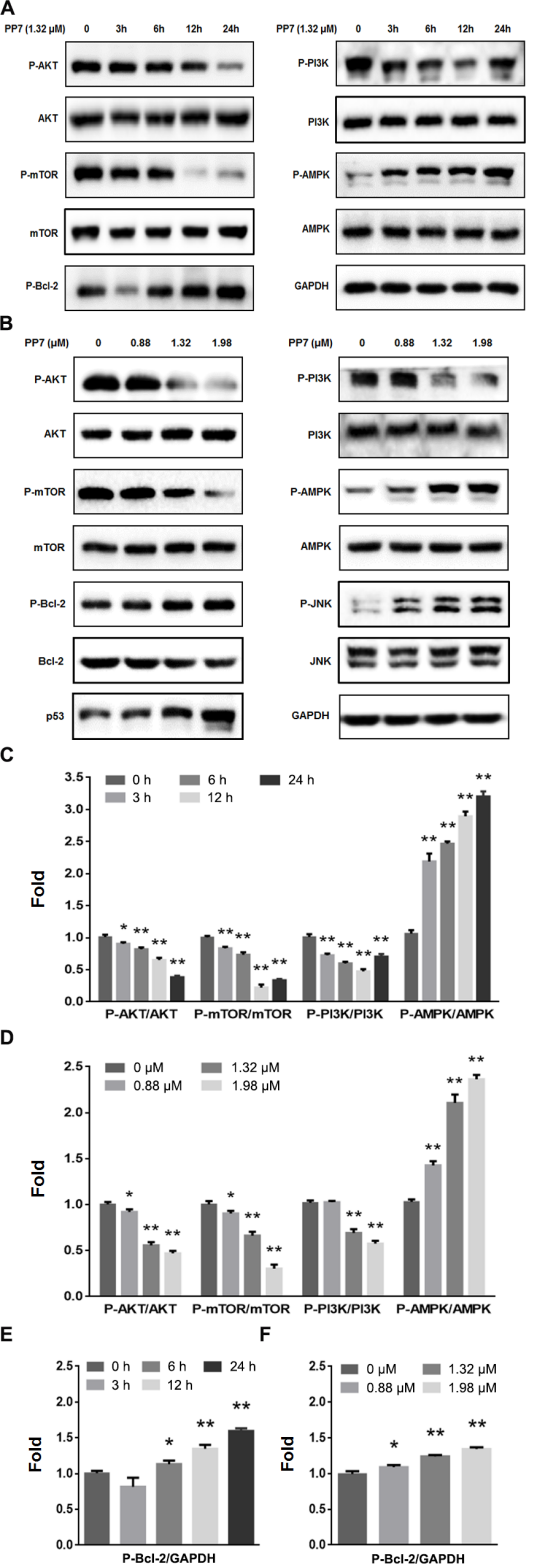
Effects of PP7 on the expression levels of components of PI3K/AKT/mTOR and JNK signaling pathways. HepG2 cells were treated with 1.32 μM PP7 for different time points (A) or the cells were treated with varying concentrations of PP7 for 24 h (B). Levels of total and phosphorylated proteins were determined by Western blot. (C), (E), (D) and (F) were densitometic analysis of (A) and (B) from three experiments, * *P* < 0.05 and ** *P* < 0.01, versus control.

In order to explore the mechanisms underlying PP7-induced autophagy, we examined the effect of PP7 on the protein level of phosphorylated mTOR, which is involved in the early triggering of autophagy [11]. Compared to the vehicle-treated control group, the levels of phosphorylated mTOR decreased by 7%, 33% and 70%, in HepG2 cells treated with 0.88, 1.32 and 1.98 μM of PP7 for 24 h, respectively (Fig 4B and 4D). The PI3K/AKT/mTOR signaling pathway is important in regulating autophagy [20]. Suppression of AKT decreases mTOR activity and promotes autophagy [45]. Thus, we evaluated whether PP7 induced autophagy is via inhibition of the PI3K-AKT-mTOR pathway. As shown in Fig 4A and 4B, the phosphorylation of both AKT and PI3K were significantly reduced in HepG2 cells treated with PP7 in a time- and dose-dependent manner, suggesting that PP7 induced autophagy in HepG2 cells is via inhibition of the phosphorylation of mTOR by the PI3K-AKT-mTOR pathway.

Recent papers have suggested that cellular stresses could promote cell death by activating AMPK-dependent autophagic death pathway. Meanwhile, AMPK-mediated mTOR inhibition also promotes autophagy [21]. We then tested whether AMPK is involved in PP7-induced autophagy in HepG2 cells. As shown in Fig 4A and 4B, when treated with PP7, the phosphorylation of AMPK was increased in an obvious time- and dose-dependent manner, suggesting that PP7 induced autophagy via inhibition of mTOR phosphorylation requires AMPK activation in HepG2 cells. Together, these results indicated that both PI3K/AKT/mTOR and AMPK/mTOR pathways mediate PP7-induced autophagy in HepG2 cells.

### Roles of JNK in PP7-induced autophagy

**Fig 5:**
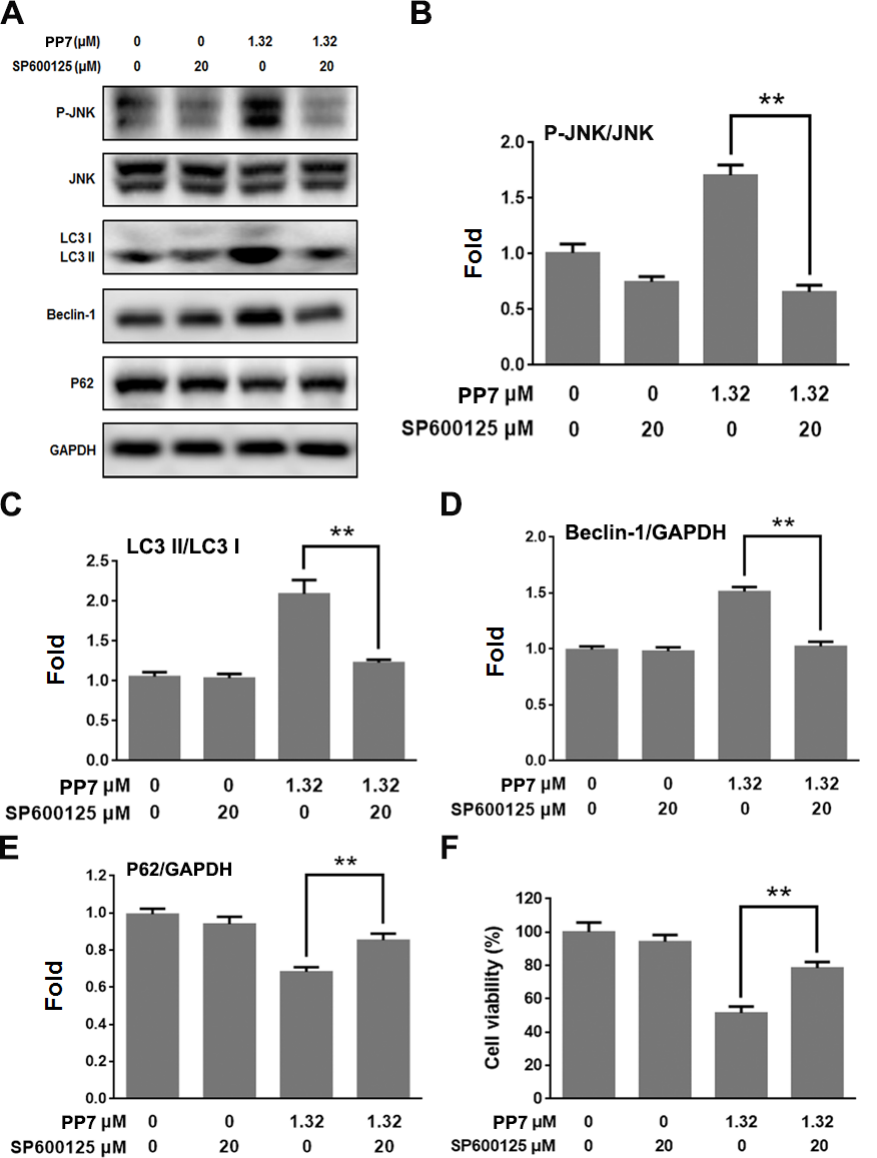
The role of JNK in PP7-triggered autophagy in HepG2 cells. (A) HepG2 cells were incubated in 1.32 μM PP7 for 24 h with or without pretreatment of JNK inhibitor SP600125 (20 μM, 1 h). The protein levels of P-JNK, JNK, P62, Beclin-1 and LC3II was detected by Western blot. (B), (C), (D) and (E) were densitometic analysis of (A) from three experiments. MTT assay was used to evaluate the viability of HepG2 cells treated by 1.32 μM PP7 for 24 h with or without pretreatment of 20 μM SP600125 for 1 h (F). Values represent the mean ± SD of at least three independent experiments. ** *P* < 0.01, compared to PP7-treated alone groups.

Various stimuli activate JNK signaling pathway, which participates in the regulation of autophagy [23]. Our results showed that PP7 increased the levels of phosphorylated JNK in a dose-dependent manner in HepG2 cells (Fig 4B). Moreover, we found that PP7 upregulated p53 protein expression and the phosphorylation of Bcl-2, and downregulated total Bcl-2 protein level in HepG2 cells (Fig 4A and 4B). To investigate the effect of JNK on the process of autophagy, cells were pretreated with JNK inhibitor SP600125 prior to being exposed to PP7. In comparison to the control group, SP600125 could significantly reduce the protein levels of Beclin-1 and LC3II, and increase the protein level of P62 in the presence of PP7. Additionally, SP600125 markedly decreased the cell death of HepG2 cells treated with PP7 (Fig 5). Taken together, these data suggested that activation of JNK and inactivation of Bcl-2 were involved in the effect of PP7 on autophagy.

## Discussion

Polyphyllin VII (PP7), a steroidal saponin, one of the main bioactive constituents of *Rhizoma Paridis*, has been identified to exert strong anticancer effects on a wide spectrum of cancers [34,35,36,37]. PP7 exhibits potential anti-HCC activities by suppression of cell proliferation and cell cycle arrest, and induction of apoptosis. Various natural chemicals have been reported to exhibit anticancer activity by triggering both autophagy and apoptosis [5,6,18,46], implicated the close relationship between autophagy and apoptosis. Previous studies reported that PP7 could induce apoptotic cell death in HepG2 cells. However, the effect of PP7 on autophagy and its role in PP7-induced cancer cell death remains unknown. In the current study, we firstly found that PP7 triggered the autophagic cell death in HepG2 cells.

Autophagy is a new target for anticancer therapy and can be activated by oxidative stress, nutrient deprivation, and chemotherapy, leading to degradation of damaged cytoplasmic proteins and organelles in response to external stressors [7,9]. During the process of autophagy, LC3I is converted to its lipidated form LC3II, which is one of the hallmarks of autophagy and essential for the formation of autophagosome [42]. P62 is used as a marker of autophagic flux and inversely correlates with autophagic activity. It binds directly to LC3 and degrades during autophagy [43]. Furthermore, examining the formation of autophagosomes and autophagic flux is also a commonly used approach in autophagy studies. Bcl-2 is critical for crosstalk between autophagy and apoptosis and it inhibits autophagy by binding to Beclin-1, which initiates autophagosome formation during autophagy [25]. In the present study, we found that PP7 treatment reduced the total Bcl-2 levels in HepG2 cells (Fig 4B), which was associated to the PP7-induced apoptosis in HepG2 cells. Our data also showed that PP7 increased the protein levels of LC3II, Beclin-1 and phosphorylated Bcl-2, while decreased the protein level of P62 in a dose- and time-dependent manner by Western blot analysis (Figs 2 and 4), indicating that PP7 treatment induced autophagy by increasing the formation of autophagosome and autophagic flux, and dissociating Beclin-1/Bcl-2 complex in HepG2 cells. Autophagy was further confirmed by the formation of GFP-LC3 puncta (Fig 2E) and autophagic vacuoles tested by MDC and AO staining (Fig 2A and 2C) induced by PP7.

The regulation of autophagy in cancer is complex. On one hand, autophagy can serve as a temporary protective mechanism for cancer cells to survive in response to certain stresses [13]. On the other hand, induction of autophagy could induce cell death, so called ‘autophagic cell death’ [10,14]. Diverse compounds can induce either pro-survival or pro-death autophagy in cancer cells [44]. In the present study, CQ co-treatment with PP7 caused down-regulation of autophagy- and apoptosis-related protein levels and decrease of the cell death of HepG2 cells (Fig 3), suggesting that PP7 induced autophagic cell death in HepG2 cells.

A number of cellular signaling pathways are involved in the regulation of autophagy. Recently, accumulating studies indicated the activation of JNK induced apoptosis and autophagy of cancer cells [23], and the JNK signaling pathway is a key requirement for up-regulating LC3 protein level [47]. Additionally, JNK also modulates the interaction between Beclin-1 and Bcl-2 [26]. JNK-dependent phosphorylation of Bcl-2 and upregulation of Beclin-1 protein level dissociates Beclin-1/Bcl-2 complex and degrade Bcl-2, leading to both apoptosis and autophagy [28,29]. JNK plays a central role both in pro-survival and pro-death autophagy. Some studies have shown that JNK was involved in the regulation of autophagic cell death of cancer cells [27,28,30]. Our results showed that phosphorylated Bcl-2 was increased (Fig 4A and 4B) and JNK was activated (Fig 4B) in HepG2 cells after PP7 treatment. Pretreatment with JNK inhibitor SP600125 suppressed JNK activity and completely prevented the induction of LC3II and Beclin-1, and the reduction of P62 by PP7 treatment (Fig 5). These results demonstrated that activation of JNK pathway by PP7 was involved in PP7-induced autophagy in HepG2 cells, which decreased the levels of Bcl-2 and promoted dissociation of Beclin-1/Bcl-2 complex. Autophagy and apoptosis may share the common upstream signals [5,23,26]. Our studies demonstrated that JNK activation was responsible for not only autophagy but also apoptosis caused by PP7. Similar results were reported that JNK activation promoted both autophagy and apoptosis by inducing Bcl-2 phosphorylation and Beclin-1 protein level, and disrupting the Bcl-2/Beclin-1 complex during nutrient starvation [26]. Park et al reported that upregulation of Beclin-1 protein level and phosphorylated Bcl-2 was involved in the JNK-modulated autophagic cell death [28]. Compared with control group, the PP7-induced protein levels of Beclin-1, P62, and LC3II were still slightly observed after pretreatment with SP600125, indicating that PP7-induced autophagy might through other pathways, such as MAPK and mTOR pathways [48], which are important regulatory pathways for autophagy.

Previous studies have shown that PP7 exerted strong anticancer activity to HepG2 cells by inhibiting PI3K/AKT pathway [34]. It is well known that in cancer cells, autophagy was negatively regulated by mTOR, which is positive mediated by PI3K/AKT pathway and a major regulator of cell growth in human cancers. Suppression of PI3K, AKT and mTOR enhances the levels of autophagy-related proteins and triggers autophagy [20]. In cancer cells, the suppression of PI3K/AKT/mTOR pathway and the upregulated protein level of Beclin-1 are consistently connected with the induction of autophagy. It is also well known that AMPK activation inhibits mTOR activity [22] and upregulates autophagic cell death [21]. In our results, treatment of PP7 up-regulated the phosphorylation of AMPK and down-regulated the phosphorylation of PI3K, AKT and mTOR in a dose- and time-dependent manner (Fig 4), indicating that PP7-induced autophagy in HepG2 cells is via inhibition of phosphorylated mTOR by the activation of AMPK and inhibition of the PI3K-AKT activity simultaneously in HepG2 cells, which is in line with previous reports [49]. Recent research reveals that the tumor suppressor protein p53 can also induce autophagy by activating AMPK to inhibit mTOR pathway [11,50]. Our results demonstrated that p53 was upregulated in a dose-dependent manner after PP7 treatment. We speculate that p53 partially mediated autophagy by inhibiting the function of PI3K/AKT/mTOR pathway. Similar results were reported that p53-mediated autophagic cell death could be induced by inhibiting PI3K/AKT pathway [51]. However, whether the activated p53 is involved in the process of autophagy remains to be further elucidated.

**Fig 6:**
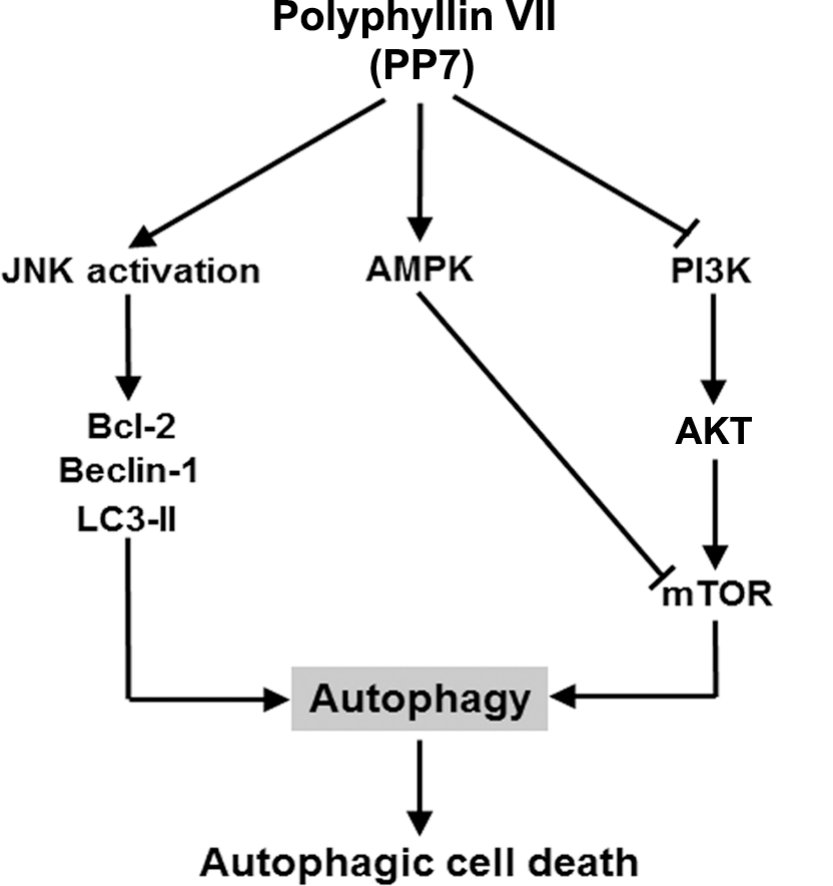
Schematic diagram showing the proposed autophagic signaling pathways triggered by PP7 in HepG2 cells.

In summary, as depicted in Fig 6, we demonstrated that PP7 induced an autophagic cell death in HepG2 cells via inhibition of PI3K/AKT/mTOR and activation of AMPK and JNK/Beclin-1/Bcl-2 pathways, suggesting that the anticancer activity of PP7 was attributable to, at least partially, the induction of autophagy and autophagic cell death, which are praised to be novel targets for cancer prevention and treatment. This study provides new insight into the molecular mechanisms of the anticancer effect of PP7 and presents that PP7 has the potential to be developed as a novel agent for the treatment of human liver cancers.

## Supporting Information

S1 Fig ^13^C NMR spectrum of Polyphyllin VII (C_5_D_5_N, 125 MHz). (DOC)

S2 Fig ^1^H NMR spectrum of Polyphyllin VII (C_5_D_5_N, 500 MHz). (DOC)

S3 Fig ESIMS spectrum of Polyphyllin VII. (DOC)

S1 Table ^13^C and ^1^H NMR data of Polyphyllin VII in C_5_D_5_N (δ in ppm). (DOC)

## Abbreviations

PP7: Polyphyllin VII
HCC: Hepatocellular carcinoma
JNK: c-Jun N-terminal kinase
AMPK: adenosine monophosphate-activated protein kinase
PI3K: phosphatidylinositol 3-kinase
AKT: protein kinase B
mTOR: mammalian target of rapamycin
mTORC1: mTOR complex 1
DMEM: Dulbecco’s modified Eagle’s medium
FCS: Fetal calf serum
CQ: chloroquine
MDC: monodansylcadaverine
AO: acridine orange
ECL: enhanced chemiluminescence
DMSO: dimethyl sulfoxide
MTT: 3-(4,5-dimethylthiazol-2-yl)-2,5- diphenyltetrazolium bromide
EGFP: enhanced green fluorescence protein
LC3: light chain 3
GFP: green fluorescence protein
P62: sequestosome-1
TCM: traditional Chinese medicine
