# Supplementary material for: Polyphyllin VII Induces an Autophagic Cell Death by Activation of the JNK Pathway and Inhibition of PI3K/AKT/mTOR Pathway in HepG2 Cells
Source: PLoS One. 2016 Jan 25;11(1):e0147405. doi: 10.1371/journal.pone.0147405 (PMC4726701; doi:10.1371/journal.pone.0147405)
Supplement: S1 Fig — (DOC) [file pone.0147405.s001.doc]

S1 Fig. ^13^C NMR spectrum of Polyphyllin VII (C_5_D_5_N, 125 MHz).
