# Supplementary material for: Polyphyllin VII Induces an Autophagic Cell Death by Activation of the JNK Pathway and Inhibition of PI3K/AKT/mTOR Pathway in HepG2 Cells
Source: PLoS One. 2016 Jan 25;11(1):e0147405. doi: 10.1371/journal.pone.0147405 (PMC4726701; doi:10.1371/journal.pone.0147405)
Supplement: S1 Table — (DOC) [file pone.0147405.s004.doc]

**S1 Table.** **^13^C** **and** **^1^H NMR data of Polyphyllin VII in C_5_D_5_N (δ in ppm).**

| aglycon | δ_C_ | δ_H_ | sugars | δ_C_ | δ_H_ |
| --- | --- | --- | --- | --- | --- |
| 1 | 37.6 | 1.69 (m), 0.94 (m) | 3-O-glc |  |  |
| 2 | 30.2 | 2.02 (m), 1.69 (m) | 1 | 100.3 | 5.00 (overlapped) |
| 3 | 78.0 | 3.83 (m) | 2 | 78.0 | 4.21 (m) |
| 4 | 39.0 | 2.75 (m) | 3 | 77.8 | 4.21 (m) |
| 5 | 140.7 | - | 4 | 77.6 | 4.34-4.46 (m) |
| 6 | 121.9 | 5.27 (d, 4.5) | 5 | 77.1 | 3.50 (m) |
| 7 | 32.5 | 1.52 (m) | 6 | 61.2 | 4.16 (br d, 11.5),  4.04 (br d, 11.5) |
| 8 | 32.4 | 1.86 (m) | 2'-O-rha |  |  |
| 9 | 50.3 | 0.90 (m) | 1 | 102.2 | 6.40 (br.s) |
| 10 | 37.2 | - | 2 | 72.5 | 4.84 (br.s) |
| 11 | 21.0 | 1.53 (m) | 3 | 72.9 | 4.64 (m) |
| 12 | 32.1 | 1.86 (m) | 4 | 74.2 | 4.34-4.46 (m) |
| 13 | 45.2 | - | 5 | 69.6 | 4.95 (m) |
| 14 | 53.1 | 2.06 (m) | 6 | 18.7 | 1.75 (d, 6.0) |
| 15 | 31.9 | 1.69 (m) | 4'-O-rha |  |  |
| 16 | 90.0 | 4.64 (dd, 9.5, 2.0) | 1 | 102.2 | 5.83 (br.s) |
| 17 | 90.2 | - | 2 | 72.9 | 4.54 (m) |
| 18 | 17.2 | 0.94 (s) | 3 | 73.4 | 4.54 (m) |
| 19 | 19.5 | 1.06 (s) | 4 | 80.5 | 4.34-4.46 (m) |
| 20 | 44.8 | 2.25 (q) | 5 | 68.3 | 4.95 (m) |
| 21 | 9.9 | 1.21 (d, 7.0) | 6 | 18.5 | 1.58 (d, 6.0) |
| 22 | 109.9 | - | 4''-O-rha -O-rha |  |  |
| 23 | 32.1 | 2.17 (m) | 1 | 103.4 | 6.28 (br.s) |
| 24 | 28.9 | 1.53 (m) | 2 | 72.7 | 4.89 (br.s) |
| 25 | 30.5 | 1.47 (m) | 3 | 73.0 | 4.54 (m) |
| 26 | 66.7 | 3.57 (d-like,9.5),  3.47 (m) | 4 | 74.1 | 4.34-4.46 (m) |
| 27 | 17.4 | 0.66 (d, 5.5) | 5 | 70.5 | 4.34-4.46 (m) |
|  |  |  | 6 | 18.9 | 1.58 (d, 6.0) |
